# Protocol for a systematic review assessing the role of digital health technology in optimising medication adherence in older patients with asthma or COPD

**DOI:** 10.1136/bmjopen-2025-105374

**Published:** 2025-11-09

**Authors:** Aseel Mahmoud, Maguy Saffouh El Hajj, Bethan Mair Treadgold, Lorna Hardy, Sumayyah Khalid, Jane Smith

**Affiliations:** 1 University of Exeter Faculty of Health and Life Sciences, Exeter, England, UK; 2 Pharmacy Department, Imperial College Healthcare NHS Trust, London, UK; 3 Clinical Pharmacy and Practice, Qatar University College of Pharmacy, Doha, Qatar; 4 Primary Care Research Group, University of Exeter Medical School, Exeter, UK; 5 University of Southampton School of Medicine, Southampton, UK; 6 Faculty of Health and Life Sciences, University of Exeter Faculty of Health and Life Sciences, Exeter, England, UK; 7 University of Manchester, Manchester, UK

**Keywords:** Asthma, Medication Adherence, Review, Frail Elderly, Chronic airways disease, Digital Technology

## Abstract

**Abstract:**

**Introduction:**

An estimated 262 million people lived with asthma globally in 2019. Similarly, in 2021, chronic obstructive pulmonary disease (COPD) was responsible for 3.5% million global deaths. They are usually distinct disorders, but the Global Initiative Chronic Obstructive Lung Disease (GOLD) 2024 strategy document asserts that asthma and COPD are conditions that may coexist in an individual and may require specific personalised approaches and treatments. It is acknowledged that they may share some common treatable traits and clinical features There are many challenges to manage asthma and COPD in the older population, including poor adherence to prescribed medications and poor inhaler techniques. The overall aim of this systematic review is to identify, appraise and synthesise available evidence around digital health interventions used to improve medication adherence in older people with asthma or COPD.

**Methods and analysis:**

This systematic review will examine studies that evaluated digital health interventions for asthma or COPD in any setting (eg, primary or secondary care). To be included, studies must be reported in English, Arabic or French and published from the year 2000 onwards. A literature search will be performed in MEDLINE via Ovid, Cochrane Central Register of Controlled Trials (CENTRAL), CINAHL, EMBASE and PsycINFO via Ovid to identify relevant articles published since 2000 and up to December 2024. No language restrictions will be applied.

The Cochrane risk-of-bias tool for randomised trials will be used to assess the quality of retrieved randomised controlled trials and quasi-experimental studies. The quality of cross-sectional, cohort and case-control studies will be assessed using the Newcastle Ottawa Scale. Mixed-methods studies will be assessed using the Mixed Methods Appraisal Tool (MMAT). The quality of qualitative studies will be assessed using the Critical Appraisal Skills Programme (CASP) qualitative checklist.

Data will be synthesised using a convergent segregated approach, which involves an independent synthesis of quantitative and qualitative data leading to the generation of quantitative and qualitative evidence, which will then be integrated.

**Ethics and dissemination:**

Ethics approval is not applicable for this study since no original data will be collected. The results will be disseminated through a peer-reviewed publication and conference presentations. Findings will be used in a bigger project aimed to answer the question on how to embed a pharmacist-led digital health service to support older people with asthma or COPD into the NHS (National Health Service) usual care.

**Prospero registration number:**

CRD42024575924.

## Introduction

 Asthma and chronic obstructive pulmonary disease (COPD) are common respiratory conditions that cause limitation of airflow in and out of the lungs.^1 2^ They are usually distinct disorders, but the Global Initiative Chronic Obstructive Lung Disease (GOLD) 2024 strategy document asserts that asthma and COPD are conditions that may coexist in an individual and may require specific personalised approaches and treatments.^3^ It is acknowledged that they may share some common treatable traits and clinical features.^4 5^ They are characterised by symptoms such as breathlessness, chest tightness, coughing and wheezing, with sudden exacerbations that can be life-threatening.^2 6 7^ As per the WHO data, in 2019, an estimated 262 million people lived with asthma globally, and due to its high prevalence, chronic nature and impacts on quality of life, asthma poses a significant health concern.^6 7^ Similarly, in 2021, COPD was responsible for 3.5% million global deaths.^2^

The ageing population is growing fast, globally,^8^ with older people being susceptible to developing long-term conditions (LTCs); in fact, 75% of people older than 75 years old have more than one LTC.^9^ With ageing, there are significant changes in lung cellular composition, with substantial anatomical and physiological changes.^10 11^ These changes may lead to a reduction in the lungs’ function and therefore, worsening of asthma and COPD symptoms in those already diagnosed with these respiratory conditions or predisposing the development of asthma and COPD.^10 11^ Despite the reported prevalence of asthma in older adults, gaps have been identified in the care they receive, and it is often underdiagnosed, misdiagnosed as COPD, undertreated and poorly self-managed.^12 13^ Also, multimorbidity may result in a poor inhaler technique among older people, diminishing treatment benefits and reducing patients’ perceptions of medication effectiveness and motivation to continue using them.^14^,^16^

Asthma and COPD are both managed with inhalers to offer short-acting or long-acting symptom relief and anti-inflammatory effects to minimise the risk of future exacerbations.^17^ Greater *adherence to asthma and COPD medications* and proper inhaler technique are related to better symptom control and reduced exacerbations, unscheduled healthcare use (hospital visits and/or admissions) and mortality.^46 18^,^21^ This may decrease asthma and COPD healthcare costs, due to fewer hospitalisations.^15 16^ The National Institute for Health and Care Excellence (NICE) identifies improved adherence as an impactful intervention for the secondary prevention of respiratory diseases and recommends research to identify cost-effective approaches to improve adherence.^18 19^

Evidence shows that there are many challenges to manage asthma and COPD in older populations that include poor adherence to prescribed medications and poor inhaler technique.^11 12 18 22^ Older people^23^ tend to have strong beliefs about medications, including concerns about side effects and effectiveness, and poor motivation for disease management due to accepting decline in functioning as part of ageing. Additionally, older people are more susceptible to cognitive impairment; thus, they may be confused as to why, how and when to use different treatments or forget to take medications. Older population is among those who might be disadvantaged by reduced access to healthcare,^24^ which may impact their ability to initiate and persist with prescribed treatments. Finally, multimorbidity may result in a poor inhaler technique among older people, diminishing treatment benefits and reducing patients’ perceptions of medication effectiveness and motivation to continue using these medications.^14^,^16^ To address these challenges, there is a need for an integrated approach to the management of asthma and COPD in older adults that uses digital tools.^13 18^

Digital tools can be convenient, easily accessed and may provide cost-effective ways of automating routine aspects of patient education, monitoring and support.^25^ Digital health interventions can increase patients’ engagement and adherence to treatment plans.^25^ For example, mobile phone apps can enhance self-management by supporting adherence, correcting inhaler technique and self-monitoring in asthma and COPD.^26^,^29^ Additionally, innovative technology has the potential to personalise management based on real-time individualised data and improve outcomes compared with non-digital interventions and usual care. For example, web‐designed education with text/call reminders^30^ has been shown to improve inhaler use in patients with asthma.

Therefore, in this review, we will focus on digital interventions in older people with asthma and COPD delivered by any healthcare professionals in any setting. However, we will focus on pharmacists and what digital interventions can be delivered by pharmacists in primary care and the community. Pharmacists play a role in asthma care globally and in the UK^31^ and are considered the most accessible healthcare profession. Pharmacists can support older patients with their medication use and inhaler technique. Additionally, they support older people with complex needs and those with a risk of inappropriate polypharmacy.^32^

There has been an increase in studies on technology-based interventions in people with asthma and COPD. There are reviews of studies that evaluated digital-based interventions in patients with asthma and COPD.^27 33^ However, these reviews have some limitations. For example, Chan *et al* focused on using maintenance therapy and only included randomised controlled trials (RCTs). There is a limited focus on interventions that are developed and targeted towards older people’s requirements for support with adherence to their asthma and COPD inhalers. Unlike other reviews, this proposed review will include all studies that targeted digital interventions provided to older people with asthma and COPD regardless of the study design including quantitative, qualitative and mixed methods studies. Moreover, it will focus on pharmacy-based interventions in the data synthesis.

### Aim and objectives

The overall aim of this systematic review is to identify, appraise and synthesise available evidence around digital health interventions used to improve medication adherence in older people with asthma or COPD.

The objectives are:

- To describe:
  - The design and target population of studies that have reported on digital interventions for older people with COPD or asthma.
  - The characteristics and components of these interventions.
- To determine:
  - The facilitators and barriers that help and/or hinder the implementation of digital health interventions in practice, with a particular focus on pharmacist-led interventions.
- To assess the effectiveness of digital health interventions in improving:
  - Patient adherence to asthma and/or COPD medications including inhalers.
  - Asthma and/or COPD outcomes including control of symptoms, quality of life or emergency healthcare use.

- Asthma and/or COPD outcomes including control of symptoms, quality of life or emergency healthcare use.

## Methods and analysis

This systematic review will be conducted using the Cochrane handbook^34^ and will be reported following the Preferred Reporting Items for Systematic Reviews and Meta-Analyses (PRISMA) guidelines.^35^

### Eligibility criteria

This systematic review will examine studies that evaluated digital health interventions for asthma or COPD in any setting (eg, primary or secondary care). To be included, studies must be reported in English, Arabic or French (studies reported in other languages will not be considered due to a lack of access to translation services). Only studies published from the year 2000 onwards will be included,^27 36^ as digital interventions implemented prior to the year 2000 are unlikely to be representative of those that are currently used to support health apps. This criterion is in line with two previous Cochrane reviews that focused on digital interventions.^27 36^

#### Type of studies

All study designs will be included, including quantitative, qualitative and mixed methods studies, including RCTs, non-controlled studies, case control and cohort studies. Only primary studies will be included. Reviews will not be included.

#### Type of participants

The review will include any studies targeting older adults aged 65 years and over with asthma or COPD (diagnosed using any recognised diagnostic criteria). This age cut-off was chosen as it represents the standard definition of older adults in the UK.^37^ Studies in which only a subset of participants meets the age inclusion criteria (for example, a mixed age group) will be included if (1) data on participants over 65 years of age are reported, or can be obtained or (2) the majority of the group (50% or more of the participants) are aged 65 years or older or their mean age is 65 years or more. Studies with a mixed sample of patients with different respiratory conditions, including COPD or asthma, will be included if data on the subgroups of interest are reported, or can be obtained or the majority are with COPD or asthma.

#### Type of interventions

Studies on any digital intervention primarily aimed at improving medication adherence will be included. A digital intervention is an electronically delivered health service or treatment, which uses technology to capture or exchange information,^38^ enable patient-clinician communication^33^ and facilitate self-management.^39^ Examples of digital health interventions in asthma/COPD include smart inhalers,^40^ mobile phone apps^41^ and web-based platforms^42^ that may target patient education, medication management or inhaler technique.^43^ For the purposes of this systematic review, digital interventions will be defined as those that are exclusively digital or include any digital element (ie, hybrid interventions). Interventions may be self-delivered by the patient, or a healthcare professional may be involved in the intervention either at the point of introduction or on an ongoing basis. The intervention may be delivered in any setting (eg, community, primary or secondary care), in any country and by any healthcare professional (including but not limited to physicians, pharmacists and nurses). Interventions delivered by pharmacists will be of particular interest.

#### Type of control

These may include usual care or non-digital intervention. In the case of qualitative or before-after studies, there may be no comparator.

#### Type of outcomes

Studies will be included if they report at least one primary outcome measure, as detailed below:

#### Primary outcomes

- Patient adherence to use of asthma or COPD medication (including inhaler(s)), as measured by any tools including via self-report,^44 45^ prescription refill data (eg, time between prescriptions^46^ or electronic monitoring^47^).
- Patient inhaler technique (quantified by the rate of wrong steps identified during inhaler use,^48^ for example).
- Outcomes including acceptability, uptake, usability and adoption of the digital intervention.

#### Secondary outcomes

- Exacerbations of asthma or COPD (eg, prompting a change in treatment, such as oral corticosteroid treatment,^49^ hospital admission or use of emergency services^50^).
- Control of asthma symptoms (measured using a validated scale such as the Asthma Control Test or Asthma Control Questionnaire,^51 52^ Asthma Quality of Life Questionnaire^53^ or St George’s Respiratory Questionnaire^54^).
- Perspectives of pharmacists, other healthcare professionals and patients in implementation and delivery of the digital intervention to the target population.

### Information sources

A literature search will be performed in the following databases to identify relevant articles published since 2000 and up to December 2024: MEDLINE via Ovid, Cochrane Central Register of Controlled Trials (CENTRAL), CINAHL, EMBASE and PsycINFO via Ovid.

### Search strategy

Search terms will include both Medical Subject Headings (MeSH) and relevant keywords (see online supplemental file 1). Search terms will be combined in the following Boolean form: ‘OR’ within concepts, and ‘AND’ across concepts, apart from asthma and COPD terms which will be combined with ‘OR’.

Each search term or combination will be searched for in the title, keywords and abstract of articles. A date filter will be applied to the search, with only studies from the year 2000 onwards included.^27 36^

### Data records and management

Studies will be exported to Rayyan software.^55^ Title and abstract screening will be undertaken independently by two researchers. Any disagreements will be discussed, and the opinion of a third member of the research team will be sought if a consensus cannot be reached. Full-text screening will also be undertaken independently by two researchers, and the same process will be followed for any disagreements. Any studies including the same intervention will be linked. Finally, citations of the included studies will be manually checked to identify further potential studies for inclusion.

Extracted data will include the study design and methods, details of the intervention, role of healthcare professionals and outcomes measured. Since we are also interested in the implementation of asthma and COPD digital interventions in practice, we will also extract any noted barriers to and/or facilitators of implementation of interventions. To ensure consistency in data extraction, the Template for Intervention Description and Replication (TIDieR) checklist and the Theme, Intensity, and Provider/Platform (TIP) framework^56^ will be used to guide the information that is being collected on each intervention, including frequency and method of delivery, setting, and use of equipment (eg, a smart inhaler). If appropriate, we will classify the interventions using the Evidence Standards Framework (EFS).^57^ ESF is developed in a dynamic way using existing literature to form a useful template to evaluate the health interventions.^57 58^ Information on the clinical data source (eg, pharmacy computer system, patient self-monitoring data, adherence measuring tool), and whether these sources are subjective or objective will also be extracted. Additionally, the specific study population eg,older adult patients with poorly controlled asthma/COPD, and participant characteristics, for example, gender, age and severity of asthma/COPD will be recorded.

### Risk of bias

The quality of included studies will be assessed independently by two members of the research team. Any disagreements will be discussed, and the opinion of a third member of the research team will be sought if a consensus cannot be reached.

The Cochrane risk-of-bias tool for randomised trials will be used to assess the quality of retrieved RCTs and quasi-experimental studies.^59^ The quality of cross-sectional, cohort and case control studies will be assessed using the Newcastle Ottawa Scale.^60^ Mixed-methods studies will be assessed using the Mixed Methods Appraisal Tool (MMAT).^61^ The quality of qualitative studies will be assessed using the Critical Appraisal Skills Programme (CASP) qualitative checklist,^62^ with scoring adapted from that developed by Butler *et al*.^63^

### Data synthesis

We will synthesise data using a convergent segregated approach as described by Cindy *et al* ^64^ in their guidance on conducting mixed methods systematic reviews, which involves an independent synthesis of quantitative and qualitative data leading to the generation of quantitative and qualitative evidence, which are then integrated together.

#### Quantitative data synthesis

We expect to identify a small number of heterogeneous interventions as studies will not be excluded based on any study design or setting. However, if a group of studies with sufficiently comparable interventions and outcomes (medication adherence to asthma or COPD medication or inhaler technique) and performed in similar settings are identified, a meta-analysis comparing interventions with comparators will be performed using a random effects meta-analysis model. RevMan software will be used to facilitate the meta-analysis. If a meta-analysis cannot be performed due to heterogeneity or the small number of included studies, a narrative synthesis will be provided in the text and supported by tables to summarise the study characteristics, interventions provided and reported results.

#### Qualitative data synthesis

Qualitative data will be synthesised using the approach described by Thomas and Harden ^65^ to explore experiences and perspectives on the facilitators and barriers that help and/or hinder the implementation of digital health interventions in practice, with a particular focus on pharmacist-led interventions. Qualitative coding will be conducted using NVivo (QSR, V.12). Two reviewers will independently code and discuss descriptive themes (based on results from the included studies). The non-adoption, abandonment, scale-up, spread, sustainability (NASSS) framework will be used to conceptualise the themes and to develop analytic themes (based on analysis by reviewers).

**STRENGTHS AND LIMITATIONS OF THIS STUDY**

- We aim to build a comprehensive review of the existing evidence on digital-based interventions for older people with asthma and chronic obstructive pulmonary disease (COPD).
- We aim to provide evidence on the current involvement of pharmacists in the provision of digital based health services for people with asthma and COPD.
- We will use a rigorous methodology in conducting the review that is based on the Cochrane handbook and will report it using the PRISMA (Preferred Reporting Items for Systematic Reviews and Meta-Analyses) checklist.
- The search strategy was developed by experienced researchers in health services research and reviewed by data synthesis experts.
- The review will include all types of studies regardless of their setting or language, which makes it comprehensive.

#### Integration of qualitative and quantitative evidence

Qualitative and quantitative data will then be integrated to produce a configured analysis to contextualise and explain the findings. This will help in assessing the effectiveness of interventions and identifying the facilitators and barriers that help and/or hinder the implementation of digital health interventions in practice in light of the experiences of participants (older people and/or healthcare practitioners). Findings will be presented as a narrative summary.

We will categorise the results separately for pharmacist-led interventions and others due to the different environment characteristics.

### Ethics and dissemination

Ethics approval is not applicable for this study since no original data will be collected. The results will be disseminated through peer-reviewed publication and conference presentations. Findings will be used in the bigger project to answer the question on how to embed a pharmacist-led digital health service to support older people with asthma or COPD into the NHS usual care.

### Patient and public involvement

Patients or the public will not be involved in the conduct of this review.

## Discussion

With the increasing number of older people, the international and national policies focus on improving older people’s health and their access to health services. Digital health technology may have a role in improving medication adherence in older people with asthma and COPD, and pharmacists can play a vital role in supporting older people to self-manage their condition.

The proposed review will update the evidence on the effectiveness of using digital health technology in older people with asthma and COPD. Moreover, it will shed light on facilitators and barriers to implementation of these interventions. Including qualitative studies will summarise perspectives of pharmacists, other healthcare professionals and patients on implementation and delivery of the digital intervention to the target population. These might be useful when developing and delivering interventions for older people with asthma and COPD. Finally, the review will help to identify possible digital interventions that pharmacists can potentially use in supporting older people with asthma and COPD.

## Supplementary material

10.1136/bmjopen-2025-105374 online supplemental file 1
