# Supplementary material for: Protocol for a systematic review assessing the role of digital health technology in optimising medication adherence in older patients with asthma or COPD
Source: BMJ Open. 2025 Nov 9;15(11):e105374. doi: 10.1136/bmjopen-2025-105374 (PMC12598943; doi:10.1136/bmjopen-2025-105374)
Supplement: online supplemental file 1 [file bmjopen-15-11-s001.docx]

| Ovid MEDLINE(R) ALL <1946 to December 10, 2024> | | |
| --- | --- | --- |
| 1 | aged.ab,hw,kw,ti. | 6243121 |
| 2 | geriatric*.ab,hw,kw,ti. | 127765 |
| 3 | elder*.ab,hw,kw,ti. | 342835 |
| 4 | old*.ab,hw,kw,ti. | 1941235 |
| 5 | aging.ab,hw,kw,ti. | 445000 |
| 6 | ageing.ab,hw,kw,ti. | 60534 |
| 7 | senior*.ab,hw,kw,ti. | 56763 |
| 8 | Pensioner*.ab,hw,kw,ti. | 1139 |
| 9 | 1 or 2 or 3 or 4 or 5 or 6 or 7 or 8 | 7658614 |
| 10 | Asthma*.ab,hw,kw,ti. | 212736 |
| 11 | Chronic respiratory condition.ab,hw,kw,ti. | 115 |
| 12 | Chronic respiratory disease.ab,hw,kw,ti. | 3132 |
| 13 | 10 or 11 or 12 | 215147 |
| 14 | COPD.ab,hw,kw,ti. | 63791 |
| 15 | Chronic obstructive pulmonary disease.ab,hw,kw,ti. | 67061 |
| 16 | Chronic obstructive lung disease.ab,hw,kw,ti. | 4829 |
| 17 | Chronic obstructive airway* disease .ab,hw,kw,ti. | 792 |
| 18 | Emphysema*.ab,hw,kw,ti. | 41029 |
| 19 | Obstructive lung diseases.ab,hw,kw,ti. | 1264 |
| 20 | (Chronic* adj3 bronchiti*).ab,hw,kw,ti. | 12176 |
| 21 | (Obstruct* adj3 (pulmonary or lung* or airway* or airflow* or bronch* or respirat*)).ab,hw,kw,ti. | 145889 |
| 22 | 14 or 15 or 16 or 17 or 18 or 19 or 20 or 21 | 192287 |
| 23 | text messag*.ab,hw,kw,ti. | 9271 |
| 24 | internet.ab,hw,kw,ti. | 139753 |
| 25 | web browser.ab,hw,kw,ti. | 2397 |
| 26 | mobile app*.ab,hw,kw,ti. | 21498 |
| 27 | Reminder systems.ab,hw,kw,ti. | 4300 |
| 28 | Telemedicine.ab,hw,kw,ti. | 56419 |
| 29 | technolog*.ab,hw,kw,ti. | 832988 |
| 30 | digital health.ab,hw,kw,ti. | 11409 |
| 31 | mobile health.ab,hw,kw,ti. | 14145 |
| 32 | website.ab,hw,kw,ti. | 28596 |
| 33 | phone*.ab,hw,kw,ti. | 70842 |
| 34 | sms.ab,hw,kw,ti. | 9043 |
| 35 | mms.ab,hw,kw,ti. | 6287 |
| 36 | e-health.ab,hw,kw,ti. | 4661 |
| 37 | telehealth.ab,hw,kw,ti. | 17414 |
| 38 | tele-health.ab,hw,kw,ti. | 336 |
| 39 | online.ab,hw,kw,ti. | 264103 |
| 40 | (app adj (smartphone* or smart-phone* or mobile* or phone* or tablet* or computer*)).ab,hw,kw,ti. | 650 |
| 41 | digital*.ab,hw,kw,ti. | 247064 |
| 42 | virtual*.ab,hw,kw,ti. | 191996 |
| 43 | ((electronic* or medication*) adj (reminder* or monitor* or record* or system* or device* or alert)).ab,hw,kw,ti. | 43320 |
| 44 | smart inhaler* .ab,hw,kw,ti. | 54 |
| 45 | m-health.ab,hw,kw,ti. | 1018 |
| 46 | tablet.ab,hw,kw,ti. | 33406 |
| 47 | remote monitoring.ab,hw,kw,ti. | 5182 |
| 48 | (short adj message*).ab,hw,kw,ti. | 1616 |
| 49 | 23 or 24 or 25 or 26 or 27 or 28 or 29 or 30 or 31 or 32 or 33 or 34 or 35 or 36 or 37 or 38 or 39 or 40 or 41 or 42 or 43 or 44 or 45 or 46 or 47 or 48 | 1718396 |
| 50 | patient acceptance of healthcare.ab,hw,kw,ti. | 33 |
| 51 | adhere*.ab,hw,kw,ti. | 298040 |
| 52 | nonadhere*.ab,hw,kw,ti. | 12304 |
| 53 | non-adhere*.ab,hw,kw,ti. | 12808 |
| 54 | complian*.ab,hw,kw,ti. | 221053 |
| 55 | non-complian*.ab,hw,kw,ti. | 8974 |
| 56 | noncomplian*.ab,hw,kw,ti. | 9846 |
| 57 | inhaler technique.ab,hw,kw,ti. | 971 |
| 58 | acceptability.ab,hw,kw,ti. | 60523 |
| 59 | uptake.ab,hw,kw,ti. | 460356 |
| 60 | usability.ab,hw,kw,ti. | 25697 |
| 61 | adoption.ab,hw,kw,ti. | 88821 |
| 62 | concordance.ab,hw,kw,ti. | 65265 |
| 63 | inhaler use.ab,hw,kw,ti. | 632 |
| 64 | 50 or 51 or 52 or 53 or 54 or 55 or 56 or 57 or 58 or 59 or 60 or 61 or 62 or 63 | 1154470 |
| 65 | 13 or 22 | 377785 |
| 66 | 9 and 49 and 64 and 65 | 787 |
| 67 | limit 66 to yr="2000 -Current" | 745 |
