# Supplementary material for: Protocol for a systematic review assessing the role of digital health technology in optimising medication adherence in older patients with asthma or COPD
Source: BMJ Open. 2025 Nov 9;15(11):e105374. doi: 10.1136/bmjopen-2025-105374 (PMC12598943; doi:10.1136/bmjopen-2025-105374)
Supplement: Supplementary file 2 [file bmjopen-2025-105374.reviewer_comments.pdf]

PEER REVIEW HISTORY

BMJ Open publishes all reviews undertaken for accepted manuscripts. Reviewers are asked to complete a checklist review form and are provided with free text boxes to elaborate on their assessment. These free text comments are reproduced below.

ARTICLE DETAILS

Title (Provisional)

Protocol for a Systematic Review Assessing the Role of Digital Health Technology in Optimising Medication Adherence in Older Patients with Asthma or COPD

Authors

Mahmoud, Aseel; El Hajj, Maguy Saffouh; Treadgold, Bethan Mair; Hardy, Lorna; Khalid, Sumayyah; Smith, Jane

---

VERSION 1 - REVIEW

**Reviewer**	**1**
**Name**	**Dilokthornsakul, Piyameth**
**Affiliation**	**Chiangmai University Faculty of Pharmacy**
**Date**	**10-Jul-2025**
**COI**	**None**

Comments on a study protocol for “Protocol for a systematic review assessing the role of digital health technology in optimizing medication adherence in older patients with asthma or COPD

General comments:

This protocol is for a systematic review of digital health technology in older patients with asthma or COPD. The study will be important for digital health in patients with asthma and COPD. The protocol is well-explained and meets the methodology standards of a systematic review. However, some suggestions should be considered.

Specific comments:

• The introduction is well presented and justifiable. However, the expected output from this study, in comparison to previous reviews, should be emphasized more.

• This statement “digital interventions implemented prior to the year 2000 are unlikely to be representative of those that are currently used to support medication adherence.” might need references to support it.

• There might be other study designs, such as self-controlled case series, etc. Will it be included?

• The definition of the majority of the group, who are aged 65+ should be provided. Cutt-off of the majority? 50%?

• The definition of digital health intervention should be clearly defined. Are adherence counters at the device and sending information to mobile devices included?

• Details on meta-analysis should be provided. What statistical model? How to assess heterogeneity and cut-off for high heterogeneity to consider not to pool them.

• Will the authors pool all digital health interventions together or separately? If separately, how will the authors classify the interventions? The TIP framework for classifying digital health interventions might help.

VERSION 1 - AUTHOR RESPONSE

No	Reviewer 1: Dr. Piyameth Dilokthornsakul, Chiangmai University Faculty of Pharmacy	Response to comments	Relevant changes in the manuscript
1	This protocol is for a systematic review of digital health technology in older patients with asthma or COPD. The study will be important for digital health in patients with asthma and COPD. The protocol is well-explained and meets the methodology standards of a systematic review. However, some suggestions should be considered.	We are glad that you found our review important and well-written. We have addressed all your suggestions below.	NA
2	The introduction is well presented and justifiable. However, the expected output from this study, in comparison to previous reviews, should be emphasized more.	We agree with your comment and elaborated on the output of the study in the introduction now. We explained that “in contrast to existing reviews, this review will focus on digital interventions that were developed and targeted to older people. Also, the review will include mixed methods and qualitative studies.” Additionally, we have clarified the output of the study in the discussion section that “our review will update the evidence on effectiveness of using digital health technology in older people with asthma and COPD. Also, it will shed the light on facilitators and barriers of implementation of these interventions in usual care.”	Introduction Page 3, L 116-120 Discussion Page 7, L282-289

3	This statement "digital interventions implemented prior to the year 2000 are unlikely to be representative of those that are currently used to support medication adherence." might need references to support it	Please see the response to comment 4 above.	Methods and analysis P4 Lines147-150
4	There might be other study designs, such as self-controlled case series, etc. Will it be included?	We have not made any exclusions based on study design and stated that all relevant primary research studies will be included.	NA
5	The definition of the majority of the group, who are aged 65+ should be provided. Cutt-off of the majority? 50%?	Thank you for your comment, we have now specified that "studies will be included if the majority of participants are older people, I e, 50% or more of total participants or if the Mean age of participants is 65 years or more"	Methods Page 4, lines 160-162
6	The definition of digital health intervention should be clearly defined. Are adherence counters at the device and sending information to mobile devices included?	Thanks for your comment, yes adherence counters and sending information on mobile devices will be included. "We have added the definition now in methods section. Digital intervention is an electronically-delivered health service or treatment, which uses technology in order to capture or exchange information , enable patient-clinician communication , and facilitate self-management , amongst other functions. Examples of digital health interventions in asthma and/or COPD include smart inhalers , mobile phone apps , and web-based platforms that may target patient education, medication management or inhaler technique. For the purposes of this systematic review, digital interventions will be defined as those that are exclusively digital or include any digital element (i.e. hybrid interventions). Interventions may be exclusively digital or include a digital element. Interventions may be self-delivered by the patient, or a healthcare professional may be involved in the intervention either at the point of introduction or on an ongoing basis."	Methods Page 4, L167-173
7	Details on meta-analysis should be provided. What statistical model? How to assess heterogeneity and cut-	Thank you for your comment. "We expect to identify a small number of heterogeneous interventions because we will not exclude any study	Methods and analysis Page 6 L245-249

	off for high heterogeneity to consider not to pool them	design or setting. However, if a group of studies with sufficiently comparable interventions and outcomes (medication adherence to asthma or COPD medication or inhaler technique) and performed in similar settings are identified, we will perform a meta-analysis comparing interventions with comparators. Meta-analysis will be performed using a random effects meta-analysis model. RevMan software will be used to facilitate the meta-analysis. In case meta-analysis is not possible due to heterogeneity and the limited number of included studies, a narrative synthesis will be provided in the text and supported by tables to summarise the study characteristics, interventions provided and reported results.”	
8	Will the authors pool all digital health interventions together or separately? If separately, how will the authors classify the interventions? The TIP framework for classifying digital health interventions might help	Thank you for your comment we added details now on classifying the interventions to the analysis section. “We will use the TIP framework to build the data extraction tool and ensure that the three themes of the framework are captured when extracting data. Additionally, we will use NICE evidence standards framework (ESF) to classify the interventions. ESF is developed in a dynamic way using existing literature to form a useful template to evaluate the health interventions”.	Methods and analysis Page 5-6, Lines 222-224

Commented [AM1]: @Smith, Jane @Treadgold, Bethan can you help please?

Commented [TB2R1]: I'm afraid being a hardcore quali researcher I can't really help with statistical model advice! I hope Jane can? apologies
